# Association of Cytomegalovirus Infection With Anti-MDA5 Antibody-Positive Dermatomyositis: A Prospective Cohort Study

**DOI:** 10.3389/fmed.2021.740154

**Published:** 2021-10-08

**Authors:** Linlin Huang, Wenbo Zhu, Yan Ye, Xiaoping Wu, Qingran Yan, Zhiqing Wang, Yanwei Lin, Sheng Chen

**Affiliations:** ^1^Department of Rheumatology, Renji Hospital, Shanghai Jiao Tong University School of Medicine, Shanghai, China; ^2^Department of Information Centre, Renji Hospital, Shanghai Jiao Tong University School of Medicine, Shanghai, China; ^3^Department of Clinical Laboratory, Renji Hospital, Shanghai Jiao Tong University School of Medicine, Shanghai, China

**Keywords:** cytomegalovirus, anti-melanoma differentiation-associated gene 5 antibody, dermatomyositis, idiopathic inflammatory myopathy, prognosis, lymphocytes subset

## Abstract

**Objectives:** To investigate whether cytomegalovirus (CMV) infection plays a role in the pathogenesis and prognosis of idiopathic inflammatory myopathy (IIM), particularly in anti-MDA5 antibody-positive (anti-MDA5^+^) dermatomyositis (DM).

**Methods:** A prospective cohort of 204 newly diagnosed IIM patients and 50 healthy individuals were enrolled in the study. CMV-IgM and CMV-IgG antibody concentrations and lymphocyte counts were analyzed. Differences in categorical data were compared using Fisher's exact test and the chi-square test. One-year survival rates were analyzed in MDA5^+^ DM patients with and without CMV infection.

**Results:** In IIM patients, the median CMV-IgM level was significantly higher than in healthy controls (6 U/mL vs. 0 U/mL, *p* < 0.05) as was the median CMV-IgG level (114 U/mL vs. 105 U/mL, *p* < 0.05). The percentage of recent CMV infections in the MDA5^+^ DM group was much higher than it was in the MDA5^−^ IIM group (19.1% vs. 7.0%, *p* = 0.009). MDA5^+^ DM patients with CMV DNA-emia had poorer 1 year survival than the CMV-DNA^−^ group (33.3% vs. 86.3%, *p* = 0.010). CMV-IgM-positive (CMV-IgM^+^) MDA5^+^ DM patients had lower CD4^+^ T cell counts (245.7 cells/μL vs. 420.5 cells/μL, *p* < 0.05) and CD19^+^ B cell counts (97.3 cells/μL vs. 240.6 cells/μL, *p* < 0.05).

**Conclusion:** The number of CMV infections was significantly higher in IIM patients, particularly in MDA5^+^ DM patients. Lower CD4^+^ T cells and CD19^+^ B cells were observed in CMV-IgM^+^ MDA5^+^ DM patients. CMV infection may have an important role in the pathogenesis and prognosis of MDA5^+^ DM by disrupting immunity.

## Key Messages

1. The prevalence of recent CMV infection was higher in IIM patients than in healthy controls, particularly in MDA5^+^ DM patients.
2. CMV-IgM^+^ MDA5^+^ DM patients had lower CD4^+^ T cell and CD19^+^ B cell counts.
3. MDA5^+^ DM patients with CMV DNA-emia had poorer 1 year survival than patients without CMV DNA-emia.

## Introduction

Idiopathic inflammatory myopathy (IIM) encompasses a group of chronic autoimmune diseases characterized by muscle damage and skin rash, including polymyositis, dermatomyositis (DM), clinically amyopathic DM (CADM), inclusion body myositis, and necrotizing autoimmune myopathy (1). Anti-MDA5 antibody-positive (MDA5^+^) DM is usually classified as CADM, which is mainly characterized by rapid progressive pulmonary interstitial disease, acute respiratory distress syndrome, and resistance to conventional treatments, resulting in a high mortality rate (2). The etiology and precipitating factors of MDA5^+^ DM remain unclear (3).

It is reported that viral infection may be involved in the occurrence of IIM, but its specific role is unknown (4, 5). A high frequency of cytomegalovirus (CMV) reactivation has been reported in DM patients, which may lead to poor morbidity and mortality (6–8). Kanetaka et al. (9) describe six cases of DM with interstitial lung disease that were improved after CMV treatment, suggesting that CMV infection was closely associated with DM. Notably, however, associations between CMV infection and MDA5^+^ DM have not been studied. Investigating whether CMV infection is closely related to the occurrence, progression, and prognosis of MDA5^+^ DM may provide insight into its etiology and treatment.

## Materials and Methods

### Study Population

From May 2017 to January 2020 a total of 204 patients at the Shanghai Renji Hospital who fulfilled the 1997 classification criteria for IIM (10) and had a disease duration of <6 months were enrolled in the current prospective cohort study. Eighty-nine were classified as having MDA5^+^ DM. All patients were followed up for at least 12 months. Fifty healthy individuals with a mean age of 52 ± 12 years were enrolled as a healthy control group (16/50 males, 32%), and MDA5^−^ IIM patients constituted a disease control group. The study was approved by the Ethics Committee of Renji Hospital, Shanghai, China (ID 2013-126), and written informed consent was provided by all participants in the study.

### Anti-CMV Antibodies, CMV-DNA, and Lymphocyte Subset Detection

Levels of anti-CMV antigen antibodies (CMV-IgM, CMV-IgG) were detected via chemiluminescence analysis (CLIA, LIAISON^®^ CMV IgG II and LIAISON^®^ CMV IgM II, DiaSorin, Italy). The normal quantitative reference range for CMV-IgM is 0–22 U/mL, and that for CMV-IgG is 0–14 U/mL. Therefore, CMV antibody-positive (CMV^+^) was defined as CMV-IgM > 22 U/mL and CMV-IgG > 14 U/mL.

CMV-DNA was detected via a PCR fluorescence probe method using the Diagnostic Kit for Quantification of Human Cytomegalovirus DNA (Sansure Biotech Inc., China). Two milliliters of peripheral venous blood were collected in an EDTA-anticoagulated tube and centrifuged at 1,600 rpm for 5 min; then 100 μL of plasma was added to 100 μL of virus enrichment solution and centrifuged at 12,000 rpm for 5 min. The supernatant was discarded, and then 50 μL of nucleic acid-releasing agent was added, and the solution was mixed. The sample was then ready to be tested. The primers used (UL123, 150 bp) were provided in the aforementioned commercial kit. CMV DNA-emia was defined as a blood CMV-DNA concentration ≥400 copies. Recent infection was defined as CMV-IgM positivity and/or CMV-DNA positivity.

Lymphocytes with different marker profiles (CD3^+^, CD3^+^CD4^+^, CD3^+^CD8^+^, CD3^+^CD19^+^, and CD3^−^CD16^+^CD56^+^) were analyzed via flow cytometry (BD MultiTEST IMK Kit, catalog# 662965, BD Biosciences, USA). Two milliliters of peripheral venous blood were collected into an EDTA-anticoagulated tube, then stored at room temperature (20–25°C). All staining was conducted within 48 h of the blood being drawn and then analyzed within 24 h of staining. For each patient sample, two BD Trucount tubes were differentially labeled, and then 20 μL of BD Multitest CD3/CD8/CD45/CD4 reagent was pipetted into the bottom of one tube, and 20 μL of BD Multitest CD3/CD16/CD56/CD45/CD19 reagent was pipetted into the bottom of the other. Fifty microliters of well-mixed anticoagulated whole blood were then pipetted into the bottom of each tube. The tubes were capped, vortexed gently, and then incubated for 15 min in the dark at room temperature (20–25°C). A 450-μL aliquot of 1X BD Multitest IMK kit lysing solution was then added to each tube, and the tubes were capped and vortexed gently. After a 15 min incubation in the dark at room temperature, the samples were analyzed via a flow cytometer and BD Multiset^TM^ software.

A total of 16 autoantibodies were detected via immunoblot testing (Euroimmun, Lubeck, Germany); anti-Mi-2α, anti-Mi-2β, anti-TIF1γ, anti-MDA5, anti-NXP2, anti-SAE1, anti-Ku, anti-PM-Scl100, anti-Scl75, anti-Jo-1, anti-SRP, anti-PL-7, anti-PL-12, anti-EJ, anti-OJ, and anti-Ro52. Antibodies were semiquantitatively analyzed via densitometry. Grayscale values ≥11 U/L were defined as positive. All procedures were conducted strictly in accordance with the manufacturer's instructions.

### Statistical Analysis

Data were analyzed using SPSS software (version 23) and GraphPad Prism software (version 8.01). Median values and interquartile ranges (IQRs) of non-normally distributed continuous data were recorded. Antibody concentrations and lymphocyte counts in two groups were compared using the Mann–Whitney U test, and differences in categorical data were compared using Fisher's exact test and the chi-square test. Kaplan–Meier curves with log rank testing were used to assess differences in survival. Two-sided *p* < 0.05 were considered statistically significant.

## Results

### Distribution of Study Populations

The study included 204 IIM patients and 50 healthy controls. In the IIM group, the mean age at onset was 53 years (standard deviation 12 years), and 33.3% were male (Table 1). IIM patients were assigned to either an MDA5^+^ DM group (34.5% males, mean age 53 ± 10 years) or an MDA5^−^ IIM group (31.5% males, mean age 51 ± 13 years) based on anti-MDA5 antibody results. Of all IIM patients, 54.41% (*n* = 111) had other myositis antibodies, and 1.96% (*n* = 4) were seronegative for myositis antibodies. In the MDA5^−^ IIM group, the antibody percentages were anti-ARS 46.09%, anti-SRP 13.91%, anti-Mi2 6.09%, anti-SAE1 3.48%, anti-NXP2 6.96%, anti-TIF1γ 21.74%, and anti-Ku 2.61%, and 3.48% were antibody-negative (see Supplementary Table 1 for more details). Demographic details, clinical manifestations, and immunological parameters of IIM patients are summarized in Table 1. Respiratory symptoms, such as coughing and shortness of breath, were more prominent in MDA5^+^ DM patients. Most of the MDA5^+^ DM patients had much higher ferritin levels than the MDA5^−^ IIM group, which may indicate a poor prognosis. MDA5^−^ IIM patients exhibited a higher prevalence of muscle conditions, such as muscle weakness and high creatine kinase levels. These observations are consistent with clinical manifestations and previous reports (11–13).

**Table 1:** IIM Patients' characteristics.

	**Total (*n* = 204)**	**MDA5^**+**^ DM (*n* = 89)**	**MDA5^**−**^ IIM (*n* = 115)**	*P*-value^*^
**Male gender, %**	33.3%	34.5%	31.5%	0.647
**Age, mean** **±** **SD**	53 ± 12	53 ± 10	51 ± 13	0.533
**Disease duration (Week, median)**	12 (8, 16)	12 (8, 16)	12 (4, 16)	0.790
**Cough**	32.6%	42.6%	21.9%	0.011
**Shortness of breath**	47.7%	64.7%	29.7%	0.001
**Muscle weakness**	51.5%	27.9%	76.6%	0.001
**Skin rash**	78.8%	94.1%	62.5%	0.001
**Arthralgia**	26.5%	32.4%	20.3%	0.117
**Creatine kinase (U/L, median)**	86 (35, 761)	47 (25, 85)	67.5 (656, 1,909)	0.001
**LDH (U/L, median)**	417 (288, 620)	347 (280, 507)	464.5 (313, 695)	0.002
**Ferritin (**μ**g/ml, median)**	597 (223, 1,500)	1,421 (596, 1,500)	343 (122, 708)	0.001
**ESR (mm/h, median)**	30 (11, 49)	25 (10, 50)	42 (26, 59.5)	0.001
**CRP (mg/L, median)**	4.7 (1.7, 9.2)	5.5 (2.0, 9.6)	4.4 (1.3, 8.4)	0.148
**Albumin (g/L, median)**	31 (28, 33)	29 (27, 32)	31 (29, 33)	0.001
**ILD (%)**	78%	96.60%	51.30%	0.001
**Therapy before admission^#^**				
Corticosteroid, mg, median	40 (15, 60)	40 (30, 60)	30 (10, 50)	0.001
0 immunosuppressive therapy drug	40.7%	33.7%	46.1%	0.074
1 immunosuppressive therapy drug	30.7%	38.2%	37.4%	0.906
2 immunosuppressive therapy drugs	14.2%	20.2%	9.6%	0.031
≥3 immunosuppressive therapy drugs	7.4%	7.9%	7.0%	0.805
Biologics	16.2%	23.6%	10.4%	0.011

*Demographic details, clinical manifestations, and immunological parameters are summarized in all IIM patients, MDA5^−^ IIM group, and MDA5^+^ DM group*.

* *A comparison of the MDA5^+^DM and MDA5^−^IIM cohorts different clinical features between the two kind of diseases*.

# *Immunosuppressive therapy drugs included Methotrexate, Azathioprine, Cyclosporine, Mycophenolate Mofetil, Tacrolimus, hydroxychloroquine, Thalidomide*.

*LDH, lactic dehydrogenase; ESR, erythrocyte sedimentation rate; CRP, C-reactive protein; ILD, interstitial lung disease*.

### CMV Antibody Levels in IIM Patients

**Figure 1:**
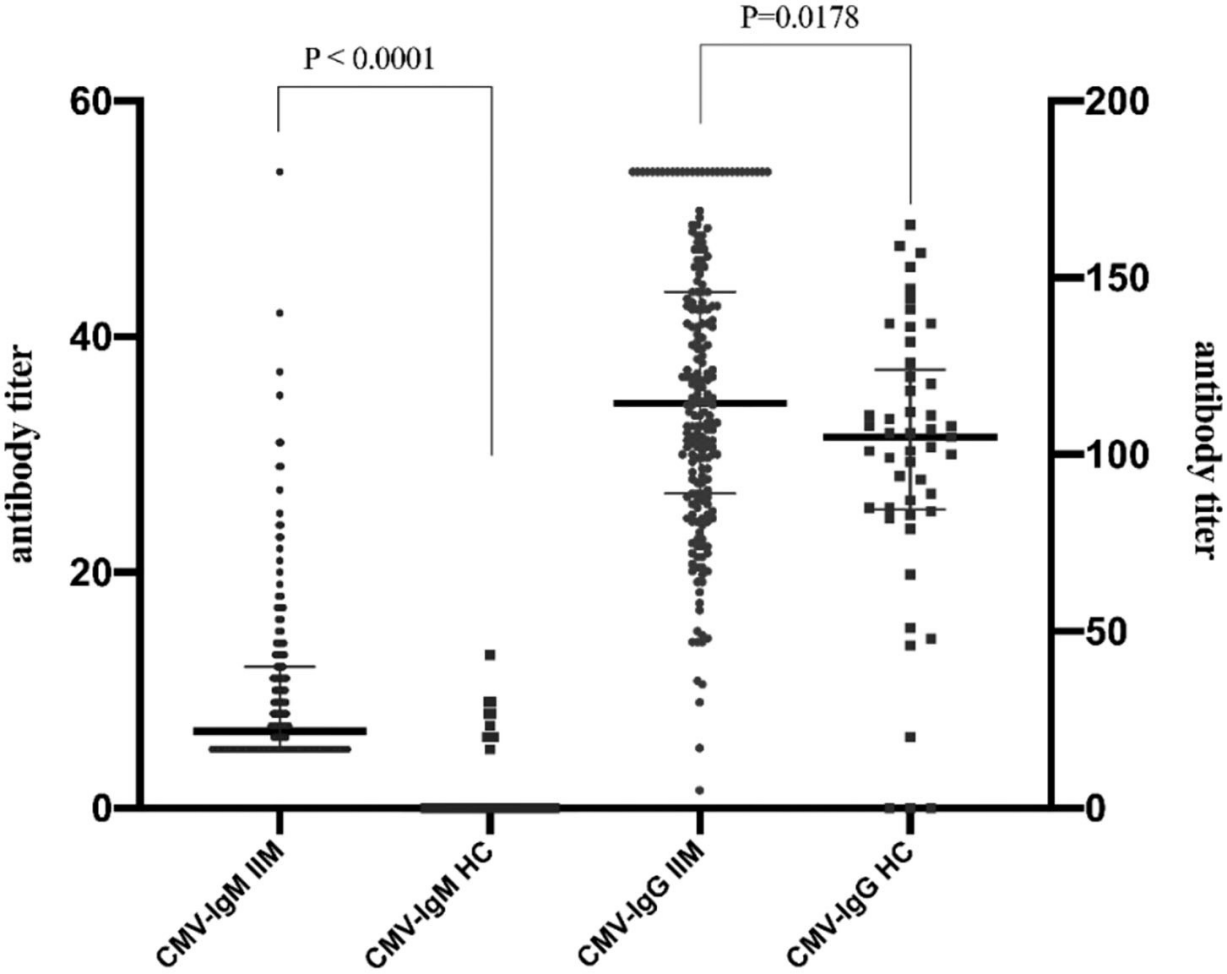
Prevalence of anti-CMV-specific antibodies in healthy controls and IIM. Higher levels of IgG and IgM antibodies against CMV were detected in IIM patients than in healthy controls (*p* < 0.05). Levels of CMV-IgM and CMV-IgG in IIM patients were significantly increased in healthy individuals compared with IIM patients [CMV-IgG: 6 U/mL, IQR (5–11) U/mL vs. 14 U/mL, IQR (89–146) U/mL, CMV-IgM: 0 U/mL vs. 105 U/mL, IQR (98–164) U/mL, both *p* < 0.05].

Higher levels of IgG and IgM antibodies against CMV were detected in IIM patients than in healthy controls (*p* < 0.05) (Figure 1). In IIM patients, the median CMV-IgM level was 6 U/mL (IQR 5–11 U/mL), and the median CMV-IgG level was 114 U/mL (IQR 89–146 U/mL), which were significantly higher than the respective values in healthy individuals (0 U/mL and 105 U/mL; both *p* < 0.05) (Table 2). The percentage of CMV-IgM^+^ participants was also significantly greater in IIM patients than in healthy controls (8.3% vs. 0%, *p* = 0.05) as was the percentage of CMV-IgG^+^ participants (99.5% vs. 93.9%, *p* = 0.024), suggesting that CMV infection was a predisposing factor in the IIM group.

**Table 2:** Distribution of CMV antibody profile in healthy individuals, IIM, MDA5^+^ DM, and MDA5^−^ IIM patients.

**Antibodies tested**	**Healthy controls (*n* = 50)**	**Total IMM (*n* = 204)**	***P*-value**	**MDA5^**+**^ DM (*n* = 89)**	**MDA5^**−**^ IIM (*n* = 115)**	***P*-value**
CMV-IgM, U/ ml	0 (0–0)	6 (5–11)	0.000^*^	8 (5–14)	6 (5–10)	0.039^*^
% positive	0%	8.3%	0.050^#^	13.5%	4.3%	0.019^#^
CMV-IgG, U/ ml	105 (84–124)	114 (89–146)	0.018^*^	116 (98–155)	112 (83–143)	0.156
% positive	93.9%	99.5%	0.024^#^	98.9%	100%	0.436

*Levels of CMV-IgM, CMV-IgG were significantly increased in IIM patients compared with healthy control groups as was the percentage of CMV-IgM^+^, CMV-IgG^+^*.

*The normal reference of quantitative CMV-IgM, CMV-IgG is 0–22, and 0–14 U/mL, respectively*.

* *P < 0.05 (Unpaired Mann–Whitney U test)*.

# *P <0.05 (Fisher's exact test/chi-square test). The level of CMV-IgM, CMV-IgG median (IQR)*.

### Recent CMV Infection in MDA5^+^ DM Patients

**Figure 2:**
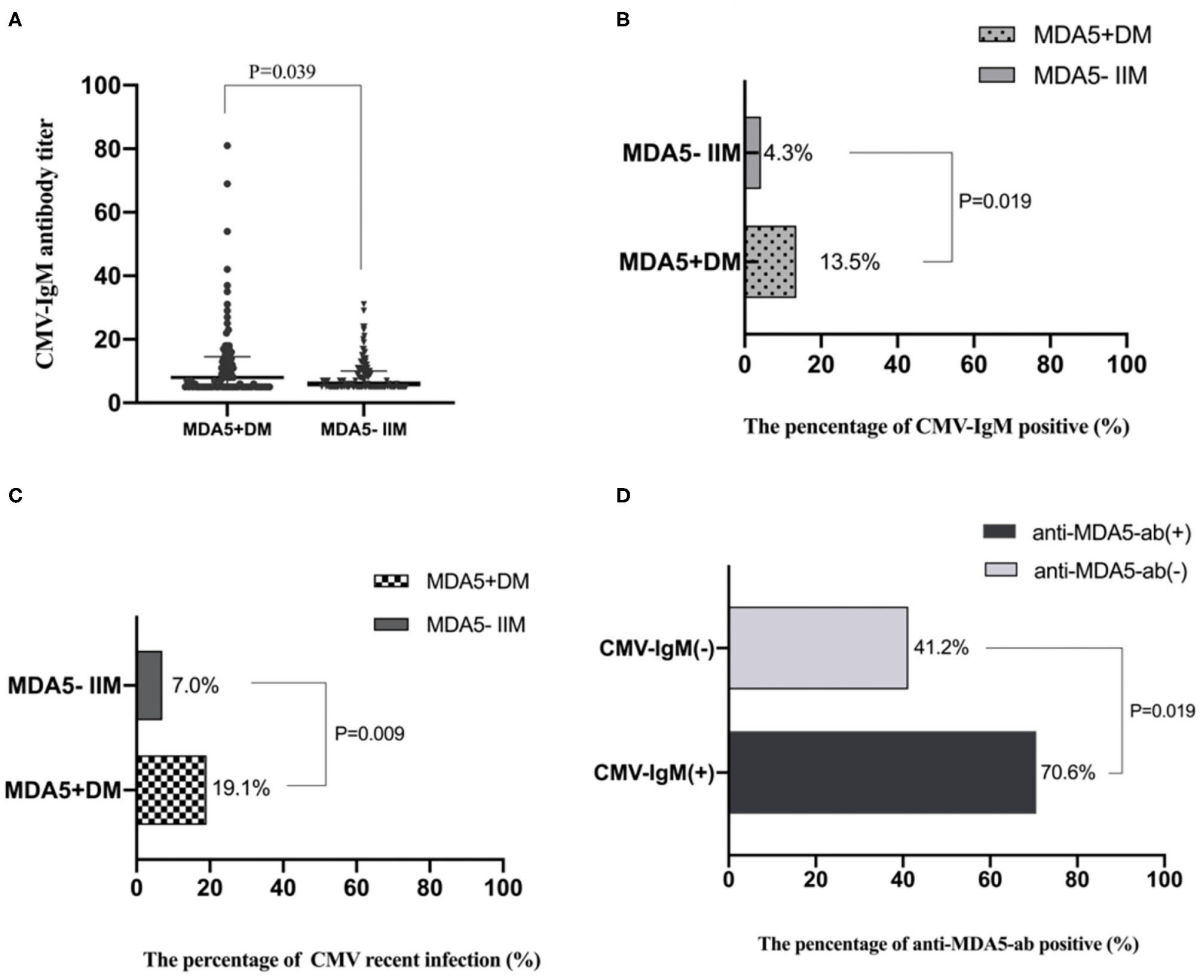
CMV infection differences between MDA5^+^DM and MDA5^−^ IIM patients. **(A)** Levels of CMV-IgM in MDA5^+^DM and MDA5^−^ IIM patients; Levels of CMV-IgM were increased in MDA5^+^DM compared with MDA5^−^ IIM patients (8 U/mL, IQR 5–14 U/mL vs. 6 U/mL, IQR 5–10 U/mL, *p* = 0.039). **(B)** The percentage of CMV-IgM^+^ in MDA5^+^DM and MDA5^−^ IIM patients; CMV-IgM^+^ rates were increased in MDA5^+^ DM patients compared with MDA5^−^ IIM patients (13.5% vs. 4.3%, *p* = 0.019). **(C)** The percentage of recent CMV infection. Recent CMV infection rates were increased in MDA5^+^ DM compared with MDA5^−^ IIM patients (19.1% vs. 7.0%, *p* = 0.009). **(D)** The percentage of anti-MDA5-ab in IIM patients with and without CMV-IgM^+^. The MDA5^+^ rates were 70.6% in CMV-IgM^+^ patients and 41.2% in CMV-IgM^−^ patients (*p* = 0.019).

Differences in CMV-IgM expression and CMV-DNA copies in MDA5^+^ DM patients and MDA5^−^ IIM patients were assessed. Levels of CMV-IgM were significantly increased in MDA5^+^ DM patients compared with MDA5^−^ IIM patients (8 U/mL, IQR 5–14 U/mL vs. 6 U/mL, IQR 5–10 U/mL, *p* = 0.039) (Figure 2A). The CMV-IgM^+^ rates were 13.5% in MDA5^+^ DM patients and 4.3% in MDA5^−^ IIM patients (*p* = 0.019) (Figure 2B). There was no significant difference in the levels of CMV-IgG between the two groups (116 U/mL, IQR 98–155 U/mL in MDA5^+^ DM patients vs. 112 U/mL, IQR 83–143 U/mL in MDA5^−^ IIM patients, *p* = 0.156).

Seven patients had CMV DNA-emia but were negative for CMV-IgM (Supplementary Table 2). The percentages of participants with recent CMV infection were 19.1% in MDA5^+^ DM patients and 7.0% in MDA5^−^ IIM patients (*p* = 0.009) (Figure 2C). Recent CMV infection rates were much higher in patients diagnosed with MDA5^+^ DM early, and the prevalence of recent CMV infection was higher in IIM patients than in healthy controls; especially in MDA5^+^ DM patients. The MDA5^+^ rates were 70.6% in CMV-IgM^+^ patients and 41.2% in CMV-IgM^−^ patients (*p* = 0.019) (Figure 2D).

### Recent CMV Infection in Patients With New-Onset IIM

Differences in CMV-IgM expression and CMV-DNA copies were compared in healthy patients and patients with new-onset IIM (*n* = 31) who had never received any steroids or immunosuppressive treatments. There were 33% of MDA5^+^ DM patients (*n* = 9) who had recent CMV infections, and 9.0% of MDA5^−^ IIM patients (*n* = 22). CMV-IgM levels were significantly higher in IIM patients than in healthy individuals (6 U/mL, IQR 5–11 U/mL vs. 0 U/mL, *p* < 0.001) (Table 3). The CMV-IgM^+^ rate was 12.9% in patients with new-onset IIM (*p* = 0.02). The rate of recent CMV infection (including CMV-IgM^+^ and CMV-DNA-emia) was 16.1% in patients with new-onset IIM (*p* = 0.007). Hence, patients with new-onset IIM without immunosuppressive treatment also had a higher prevalence of recent CMV infection, suggesting that CMV infection may participate in the pathogenesis of IIM as an environmental induction factor, particularly in MDA5^+^ DM patients.

**Table 3:** Distribution of CMV antibody profile in healthy individuals, new-onset IIM patients.

**Antibodies tested**	**Healthy controls (*n* = 50)**	**newly onset IMM (*n* = 31)**	***P*-value**
CMV-IgM, U/ ml^†^	0 (0–0)	6 (5–11)	0.000^*^
% positive	0%	12.90%	0.020^#^
CMV-IgG, U/ ml^†^	105 (84–124)	99 (74–136)	0.778^*^
% positive	93.90%	100.00%	0.279^#^
Recent CMV infection	0%	16.10%	0.007^#^
% positive			

*Levels of CMV-IgM were significantly increased in new-onset IIM patients compared with healthy control groups as was the percentage of CMV-IgM^+^ and recent CMV infection*.

*The normal reference of quantitative CMV-IgM, CMV-IgG is 0–22, and 0–14 U/mL, respectively*.

* *Unpaired Mann–Whitney U test*.

# *Fisher's exact test/chi-square test*.

† *The level of CMV-IgM, CMV-IgG median (IQR)*.

### Survival Analysis

**Figure 3:**
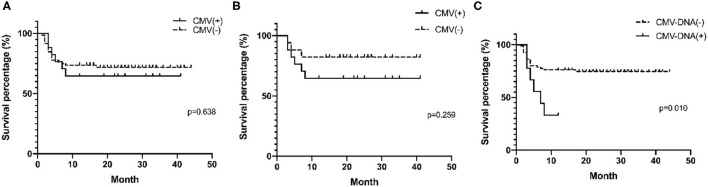
Survival analysis of MDA5^+^ DM patients with CMV infection by Kaplan–Meier curve. **(A)** Survival percentages stratified to recent CMV infection (CMV-IgM positive and/or CMV DMA-emia) in all MDA5^+^DM patients (*p* = 0.638). **(B)** Survival percentages stratified to recent CMV infection (CMV-IgM positive and/or CMV DMA-emia) in postmatched MDA5^+^ DM patients (*p* = 0.259). **(C)** Survival percentages stratified to CMV DMA-emia in all MDA5^+^ DM patient (*p* = 0.010). One-year-survival percentages were determined by Kaplan–Meier curves with log rank testing.

Prognoses were compared in MDA5^+^ DM patients with and without recent CMV infection as determined by CMV-IgM positivity and/or blood CMV-DNA ≥400 copies (CMV DNA-emia) (Figure 3A). The two survival curves cross many times, indicating that other factors affect the prognosis of MDA5^+^ DM (further details are presented in Supplementary Table 3). To minimize the baseline data imbalance, the long-term outcome in MDA5^+^ DM patients with recent CMV infection was mainly assessed in postmatched patients. Matched factors included age, symptoms, disease duration, initial treatment, interstitial lung disease (ILD), creatine kinase, erythrocyte sedimentation rate, and ferritin. Clinical features of postmatched patients at baseline are summarized in Supplementary Table 4. The cumulative survival rate at 12 months was lower in CMV-IgM^+^ than in CMV-IgM^−^ patients (64.7% vs. 82.4%, *p* = 0.259), but the difference was not statistically significant (Figure 3B). MDA5^+^ DM patients with CMV DNA-emia tended to have poorer long-term outcomes than MDA5^+^ DM patients without CMV DNA-emia (33.3% vs. 86.3%, *p* = 0.010) (Figure 3C).

### Lymphocyte Subsets in MDA5^+^ DM Patients With CMV

**Figure 4:**
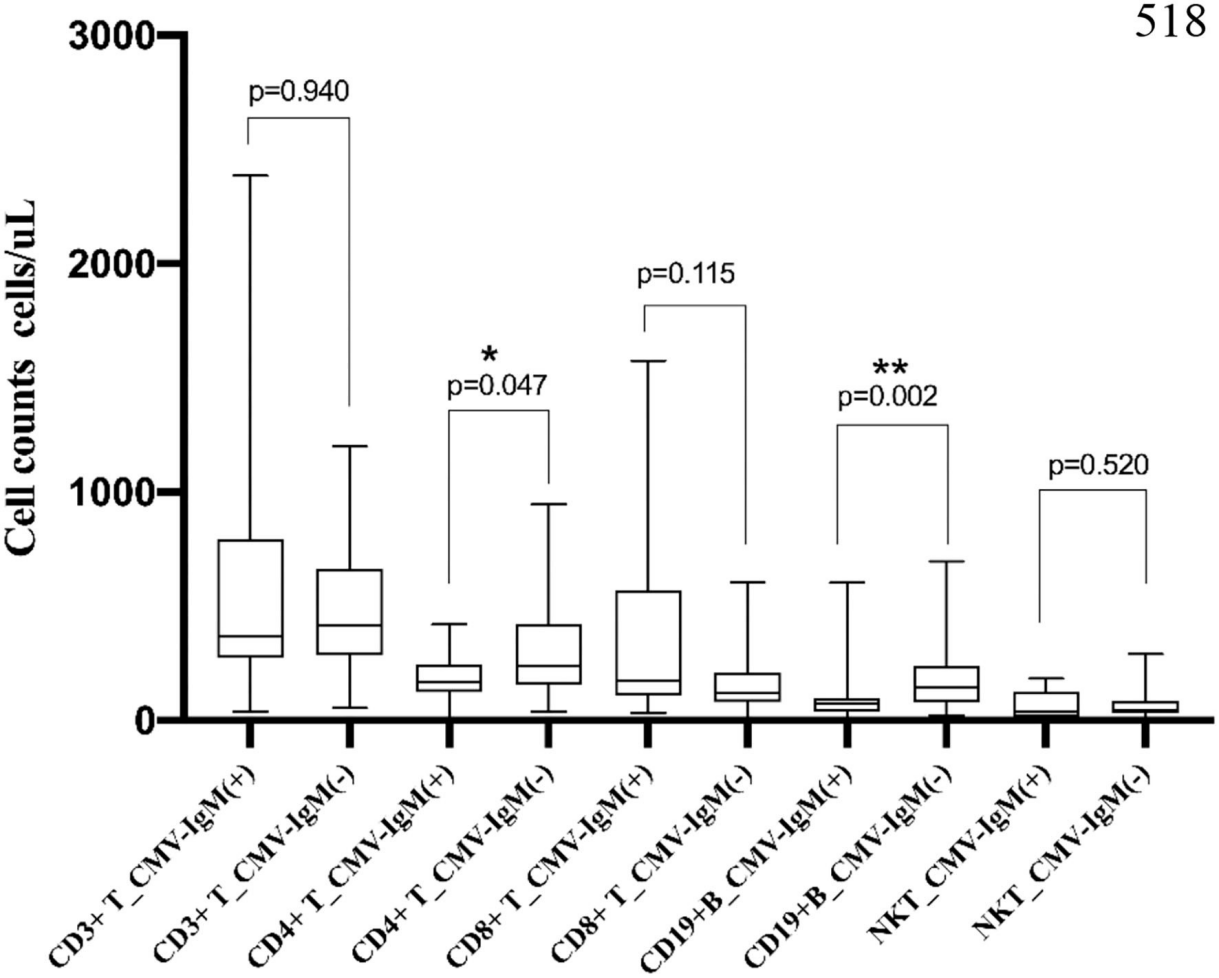
Association of lymphocyte subset and CMV-IgM in MDA5 + DM. CD4^+^ T cell counts were significantly decreased in CMV-IgM^+^ group compared with CMV-IgM^−^ group (245.7 cells/μL, IQR 167.1–374.1 cells/μL vs. 420.6 cells/μL, IQR 238.1–603.4 cells/μL, *p* = 0.047). CD 19+B cell counts was also much lower in CMV-IgM^+^ group than CMV-IgM^−^ group (420.6 cells/μL, IQR 238.1–603.4 cells/μL vs. 240.6 cells/μL, IQR 146.4–372.2 cells/μL, *p* = 0.006) cells/ul, *p* = 0.006). *P < 0.05, **P < 0.01.

In the MDA5^+^ DM group, 12 patients (13.5%) were CMV-IgM^+^ (Figure 4). CD4^+^ T cell counts were significantly lower in the CMV-IgM^+^ group than in the CMV-IgM^−^ group (245.7 cells/μL, IQR 167.1–374.1 cells/μL vs. 420.6 cells/μL, IQR 238.1–603.4 cells/μL, *p* = 0.047). The number of CD19^+^ B cells was also much lower in the CMV-IgM^+^ group than in the CMV-IgM^−^ group (420.6 cells/μL, IQR 238.1–603.4 cells/μL vs. 240.6 cells/μL, IQR 146.4–372.2 cells/μL, *p* = 0.006). CD8^+^ T cell levels and natural killer cell levels were evidently little affected by CMV infection. Thus, CMV infection may disturb the immune system in MDA5^+^ DM patients by decreasing CD4^+^ T cells and B cells.

## Discussion

The hallmark of MDA5^+^ DM is auto-antibodies targeting MDA5, which is characterized by the presence of a DM-specific rash (also known as periorbital rash or Gottron's rash), ILD, and ultimately respiratory failure (14). The pathogenesis of MDA5^+^ DM is currently unclear. MDA5 is a widely expressed cytosolic RNA helicase that can be activated by RNA viruses (15). The association between viral infection and MDA5^+^ DM warrants further investigation.

CMV is reportedly a major cause of morbidity and mortality in patients with impaired immunity, such as HIV patients and those with autoimmune diseases (16). In 2006, Hashimoto et al. (17) described the cases of two myositis patients infected with CMV to highlight the importance of CMV in the progression of DM, and proposed the importance of close monitoring of such patients and rapid initiation of appropriate treatment to manage suspected CMV infection. The possible mechanisms of autoimmunity disorder triggered by viral infection may include molecular mimicry (18, 19), or the virus itself may activate antigen-presenting cells directly, in turn, leading to the activation of T cells, resulting in a cytokine storm and ultimately tissue damage (20).

The current study is the first to report the prevalence of recent CMV infection in patients with newly diagnosed MDA5^+^ DM and the first to investigate the clinical significance of CMV infection in the pathogenesis and prognosis of MDA5^+^ DM patients. Whether or not CMV-IgM false-positive results were caused by interfering infections with Epstein–Barr virus (EBV), hepatitis E virus, and rheumatoid factor (RF), IgM positivity was also considered in the study. No CMV-IgM^+^ patients were positive for hepatitis E virus or RF IgM. Five patients were positive for EBV early antigen IgG, and only one of these was positive for EBV viral capsid antigen IgM. Given that all five were positive for CMV-IgG, we surmised that CMV-IgM false positivity was less likely in these patients. There were also MDA5^+^ DM patients who had never had CMV infection (1.1% of patients were CMV-IgG^−^), indicating that other factors may also participate the pathogenesis of MDA5^+^ DM (21, 22).

Whether recent CMV infection and, particularly, CMV DNA-emia were associated with poor survival in MDA5^+^ DM patients was investigated in the present study. Several previous studies indicate that CMV infection is associated with disease activity (9, 23). In the current IIM cohort, patients with recent CMV infections tended to have poorer outcomes, but the trend was not statistically significant, which may be due to the small number of patients or prompt antiviral therapy (ganciclovir). The high mortality in MDA5^+^ DM patients with CMV DNA-emia (66.7%, *n* = 8) warrants extensive attention.

Disrupted proportions of lymphocyte subsets were observed in CMV-IgM^+^ MDA5^+^ DM patients in the current study, including lower CD4^+^ T cells and CD19^+^ B cells in all MDA5^+^ patients (*n* = 89). The number of MDA5^+^ DM patients who did not receive any therapy (*n* = 9, CMV-IgM^+^ *n* = 6, CMV-IgM^−^ *n* = 3) was too small to facilitate informative lymphocyte subset analysis; thus, we performed the same analysis in postmatched MDA5^+^ DM patients (*n* = 34; see Supplementary Table 3 for further details). CD4^+^ T cell counts were significantly lower in CMV^+^ patients than in CMV^−^ participants (164.2 cells/μL, IQR 117.7–212.8 cells/μL vs. 259.6 cells/μL, IQR 196.9–434.3 cells/μL, *p* = 0.014). CD19^+^ B cell counts were also lower in CMV^+^ patients than in CMV^−^ participants (91.5 cells/μL, IQR 51.3–147.8 cells/μL vs. 159.35 cells/μL, IQR 83.5–253.8 cells/μL, *p* = 0.113) although the difference was not statistically significant. This is consistent with a recent report that CD4^+^CXCR4^+^ T cells were associated with the severity of MDA5^+^ DM-ILD and mortality in a study in which high-resolution computed tomography scores and pulmonary functions (forced vital capacity and diffusion capacity of the lung for carbon monoxide) were evaluated (24). In recent studies, circulating CD28-null T cells were also significantly higher in CMV^+^ DM patients, in whom CD28-null T cells may accelerate inflammation in muscle (25). As well as T cells, percentages of CD19^+^ B cells were reduced in MDA5^+^ DM patients in the present study. Researchers speculate that virus-induced activation of antigen-presenting cells, particularly plasmacytoid dendritic cells, may contribute to T cell-independent activation of B cells and the generation of autoimmune phenomena (19). Treatment with the CD20-targeting monoclonal antibody rituximab has been applied in rapid progressive pulmonary interstitial disease patients who were refractory to conventional treatments (26). Further details of the mechanisms involved provide insight into better treatments.

The present study had several limitations. All patients enrolled were from a single center, and the number of CMV-infected patients was limited. Further multicenter registered database and potential pathogenesis investigations need to be conducted. The prognosis of MDA5^+^ DM patients may be affected by many factors, particularly disease activity, drug therapy, and immune status. There may also have been some statistical bias in the survival analysis.

## Conclusion

A higher incidence of CMV infection was observed in IIM patients, indicating that CMV may act as an environmental factor that predisposes patients to the development of IIM. Associations between CMV-IgM antibody levels may contribute to the role of CMV in the pathogenesis of MDA5^+^ DM by regulating lymphocyte subsets, such as decreasing CD4^+^ T cells and CD19^+^ B cells. Therefore, we suggest that it is necessary to screen patients for CMV infection via the detection of CMV antibodies and CMV DNA, which can be used as prognostic markers in MDA5^+^ DM patients. Timely antiviral therapy may also improve prognoses. Further studies are required to confirm the predictive value of CMV infection.

## Data Availability Statement

The original contributions generated for the study are included in the article/Supplementary Material, further inquiries can be directed to the corresponding author/s.

## Ethics Statement

The studies involving human participants were reviewed and approved by the Ethics Committee of Renji Hospital, Shanghai, China. The patients/participants provided their written informed consent to participate in this study.

## Author Contributions

LH, YL, and SC participated in study conception, design, and supervision. LH, WZ, YY, XW, QY, ZW, YL, and SC were involved in literature searches, study design, data collection, statistical analysis, interpretation, and writing and revision of the manuscript. All authors revised the manuscript and approved the final report.

## Funding

This work was supported by the National Natural Science Foundation of China (Grant Number: 81771752).

## Conflict of Interest

The authors declare that the research was conducted in the absence of any commercial or financial relationships that could be construed as a potential conflict of interest.

## Publisher's Note

All claims expressed in this article are solely those of the authors and do not necessarily represent those of their affiliated organizations, or those of the publisher, the editors and the reviewers. Any product that may be evaluated in this article, or claim that may be made by its manufacturer, is not guaranteed or endorsed by the publisher.
