# Supplementary material for: Association of Cytomegalovirus Infection With Anti-MDA5 Antibody-Positive Dermatomyositis: A Prospective Cohort Study
Source: Front Med (Lausanne). 2021 Oct 8;8:740154. doi: 10.3389/fmed.2021.740154 (PMC8531210; doi:10.3389/fmed.2021.740154)
Supplement: Supplementary file 1 [file Table_1.DOCX]

**Online supplementary**

**Supplementary table S1. The prevalence of myositis antibodies in MDA5^-^IIM group.**

**Supplementary table S2. Details of clinical features of the 25 cases with CMV infection at diagnosis.**

**Supplementary table S3. Patients’ characteristics of MDA5^+^DM with and without recent CMV infection**. The significant difference of LDH between the two groups which may affect survival differences. (*p* < 0.05)

**Supplementary table S4. Patients’ characteristics of recent CMV infection 1:1 post-matched in MDA5^+^DM to analyze the 12-month survival rate**. Post-matched patients similar in ages, disease duration, as well as experimental result and clinical data.

**Supplementary table S1.** The prevalence of myositis antibodies in MDA5^-^IIM group.

|  | Percentages | Numbers |
| --- | --- | --- |
| anti-ARS antibodies* | 46.09% | 53 |
| anti-SRP antibodies | 13.91% | 16 |
| anti-Mi2 antibodies | 6.09% | 7 |
| anti-SAE1 antibodies | 3.48% | 4 |
| anti-NXP-2 antibodies | 6.96% | 8 |
| anti-TIF1-Gamma antibodies | 21.74% | 25 |
| anti-Ku antibodies | 2.61% | 3 |
| antibody-negative | 3.48% | 4 |

*anti-ARS antibodies include anti-PM-Scl100, anti-Scl75, anti-Jo-1, anti-PL-7, anti-PL-12, anti-EJ, anti-OJ

**Supplementary table S2.** Details of clinical features of the 25 cases with CMV infection at diagnosis

| Num | Diagnosis | Antibody | Duration | Gender | Age | ILD | CMV-IgG | CMV-IgM | CMV-DNA | Corticosteroid | Immunosuppressants | Ganciclovir | Followed time | Outcome |
| --- | --- | --- | --- | --- | --- | --- | --- | --- | --- | --- | --- | --- | --- | --- |
| 1 | ADM | anti-MDA5^-^ab | 3 | male | 50 | 1 | 104 | 17 | 180 | 100 | HCQ，CsA | Yes | 12 | survive |
| 2 | ADM | anti-MDA5^-^ab | 2 | male | 47 | 1 | 102 | 10 | 25400 | 100 | CsA | Yes | 3 | dead |
| 3 | ASS | anti-EJ-ab | 1 | Female | 68 | 1 | 99 | 5 | 1670 | 50 | none | Yes | 12 | survive |
| 4 | DM | anti-MDA5^-^ab | 5 | Female | 44 | 1 | 180 | 35 | 400 | 100 | HCQ | No | 25 | survive |
| 5 | DM | anti-MDA5^-^ab | 3 | Female | 43 | 1 | 180 | 29 | 400 | 100 | CYC | No | 19 | survive |
| 6 | PM | anti-NXP2 -ab | 2 | male | 62 | 0 | 180 | 24 | 10200 | 15 | TwHF | Yes | 14 | survive |
| 7 | ADM | anti-TIF1r-ab | 1 | male | 58 | 1 | 107 | 29 | 400 | 100 | None | No | 16 | survive |
| 8 | DM | anti-MDA5^-^ab | 5 | Female | 61 | 1 | 180 | 54 | 400 | 100 | CYC | Yes | 33 | survive |
| 9 | DM | anti-MDA5^-^ab | 3 | Female | 63 | 1 | 165 | 5 | 46100 | 150 | CsA,Tofa,Thd | Yes | 12 | survive |
| 10 | ADM | anti-MDA5^-^ab | 3 | male | 60 | 1 | 137 | 31 | 400 | 100 | None | Yes | 23 | survive |
| 11 | PM | anti-PL-7-ab | 1 | male | 58 | 1 | 130 | 16 | 1110 | 75 | Tak | Yes | 12 | survive |
| 12 | ADM | anti-MDA5^-^ab | 4 | Female | 42 | 1 | 180 | 69 | 400 | 100 | MMF,HCQ | No | 22 | survive |
| 13 | DM | anti-TIF1-γ+ | 2 | Female | 60 | 0 | 180 | 10 | 641000 | 100 | HCQ | Yes | 6 | dead |
| 14 | DM | anti-MDA5^-^ab | 4 | Female | 68 | 1 | 109 | 81 | 141000 | 150 | MMF,Tak | Yes | 5 | dead |
| 15 | DM | anti-MDA5^-^ab | 6 | Female | 60 | 1 | 180 | 42 | 400 | 100 | CsA | No | 41 | survive |
| 16 | ADM | anti-MDA5^-^ab | 1 | Female | 48 | 1 | 111 | 22 | 400 | 100 | None | No | 35 | survive |
| 17 | ASS | anti-J0-1-ab | 2 | Female | 60 | 1 | 115 | 31 | 400 | 100 | MTX | No | 42 | survive |
| 18 | ADM | anti-MDA5^-^ab | 3 | Female | 63 | 1 | 114 | 5 | 1180 | 50 | Tak,Tofa,CYC | Yes | 12 | survive |
| 19 | DM | anti-SRP-ab | 3 | Female | 21 | 0 | 180 | 24 | 1080 | 50 | CsA | Yes | 38 | survive |
| 20 | DM | anti-MDA5^-^ab | 1 | Female | 54 | 1 | 101 | 27 | 905 | 50 | Tak | Yes | 7 | dead |
| 21 | DM | anti-MDA5^-^ab | 2 | male | 53 | 1 | 117 | 23 | 400 | 100 | HCQ,MTX,CsA | No | 43 | survive |
| 22 | ADM | anti-MDA5^-^ab | 6 | male | 43 | 1 | 180 | 25 | 1240 | 100 | None | Yes | 8 | dead |
| 23 | DM | anti-MDA5^-^ab | 5 | Female | 62 | 1 | 180 | 37 | 400 | 100 | None | No | 31 | survive |
| 24 | ADM | anti-MDA5^-^ab | 3 | Female | 55 | 1 | 180 | 17 | 470 | 75 | MMF,HCQ,Thd | No | 4 | dead |
| 25 | ADM | anti-MDA5^-^ab | 2 | male | 47 | 1 | 180 | 23 | 10100 | 150 | CsA | Yes | 3 | dead |

**Supplementary table S3. Patients’ characteristics of MDA5^+^DM with and without recent CMV infection**.

|  | CMV（+）† | CMV（-） | P |
| --- | --- | --- | --- |
|  | （n=17） | （n=72） |  |
| Male gender | 29.40% | 31.90% | 0.840 |
| Onset age, mean±SD | 54(46,62) | 54(47,62) | 0.884 |
| Disease duration(month, median) | 3(3,5) | 3(2,4) | 0.091 |
| Cough | 36.40% | 43.90% | 0.747 |
| Shortness of breath | 63.60% | 64.90% | 1.000 |
| Muscle weakness | 27.30% | 27.30% | 1.000 |
| Skin rash | 100% | 94.70% | 0.515 |
| Arthralgia | 27.30% | 28.10% | 1.000 |
| Creatine kinase, U/L, median | 44(24,69) | 47.5(26.25,86) | 0.635 |
| ESR, mm/hr, median | 58(31,66) | 40(24,54) | 0.073 |
| Creatinine, μmmol/L, median | 40(33,60) | 46(38,60) | 0.261 |
| Ferritin, μg/ml, median | 1342(622,1500) | 1500(595,1500) | 0.742 |
| LDH,U/L, median | 447(356,586) | 332(268,456.25) | 0.020 |
| ILD(%) | 100.0% | 96.5% | 1.000 |
| Therapy* |  |  |  |
| Corticosteroid, mg, median | 100(50,150) | 100(50,100) | 0.638 |
| 0 immunosuppressive therapy drug | 29.40% | 37.50% | 0.532 |
| 1 immunosuppressive therapy drug | 35.30% | 38.90% | 0.784 |
| 2 immunosuppressive therapy drugs | 24% | 16.70% | 0.497 |
| ≥3 immunosuppressive therapy drugs | 11.80% | 6.90% | 0.615 |
| Biologics | 11.80% | 6.90% | 0.615 |

***** Immunosuppressive therapy drugs included Methotrexate, Azathioprine, Cyclosporine, Mycophenolate Mofetil, Tacrolimus, hydroxychloroquine,Thalidomide.

†CMV(+) included MDA5^+^DM patients with CMV-IgM posive and/or CMV DNA-enimia

LDH：lactic dehydrogenase； ESR：erythrocyte sedimentation rate；

CRP：C-reactive protein； ILD：Interstitial Lung Disease

**Supplementary table S4. Patients’ characteristics of recent CMV infection 1:1 post-matched in MDA5^+^DM to analyze the 12-month survival rate**.

|  | CMV（+）† | CMV（-） | P |
| --- | --- | --- | --- |
|  | （n=17） | （n=17） |  |
| Male gender | 29.4% | 29.4% | 1.000 |
| age, median | 55(49,64) | 54(46,62) | 0.474 |
| Disease duration(month, median) | 3(3,5) | 3(3,5) | 0.792 |
| Cough | 36.4% | 27.3% | 1.000 |
| Shortness of breath | 63.6% | 45.5% | 0.670 |
| Amyasthenia | 27.3% | 27.3% | 1.000 |
| Skin rash | 100.0% | 90.9% | 1.000 |
| Arthralgia | 27.3% | 63.5% | 0.198 |
| CK, U/L, median | 39.85(33,60) | 46(34,52) | 0.704 |
| ESR, mm/hr, median | 57(30,65) | 33(26,48) | 0.193 |
| Creatinine, μmmol/L | 40(33,59) | 46(34,52) | 0.759 |
| Ferritin, μg/ml, median | 1342(622,1500) | 1500(920,1500) | 0.296 |
| LDH, U/L, median | 447(356,586) | 313(252,531) | 0.176 |
| ILD (%) | 100% | 100% | 1.000 |
| Therapy* |  |  |  |
| Corticosteroid, mg, median | 100(50,150) | 100(50,150) | 1.000 |
| 0 immunosuppressive therapy drug | 29.4% | 29.4% | 1.000 |
| 1 immunosuppressive therapy drug | 41.2% | 35.3% | 1.000 |
| 2 immunosuppressive therapy drugs | 23.5% | 29.4% | 1.000 |
| ≥3 immunosuppressive therapy drugs | 11.8% | 0.0% | 1.000 |
| Biologics | 11.8% | 5.9% | 0.485 |

***** Immunosuppressive therapy drugs included Methotrexate, Azathioprine, Tacrolimus, Cyclosporine, Mycophenolate Mofetil, hydroxychloroquine, Thalidomide.

† CMV (+) included MDA5^+^DM patients with CMV-IgM positive and/or CMV DNA-emia

LDH：lactic dehydrogenase； ESR：erythrocyte sedimentation rate；

CRP：C-reactive protein； ILD：interstitial Lung Disease
